# Algorithm-assisted diagnosis of Hirschsprung’s disease – evaluation of robustness and comparative image analysis on data from various labs and slide scanners

**DOI:** 10.1186/s13000-024-01452-x

**Published:** 2024-02-06

**Authors:** Ariel Greenberg, Benzion Samueli, Shai Farkash, Yaniv Zohar, Shahar Ish-Shalom, Rami R. Hagege, Dov Hershkovitz

**Affiliations:** 1 Institute of Pathology, Tel-Aviv Sourasky Medical Center, 6 Weizmann Street, 6423906 Tel Aviv, Israel; 2 Department of Pathology, Soroka University Medical Center, 76 Wingate Street, 8486614 Be’er Sheva, Israel; 3 Department of Pathology, Emek Medical Center, Yitshak Rabin Boulevard 21, 1834111 Afula, Israel; 4 Department of Pathology, Rambam Medical Center, 8 Haalia Hashnia, 3525408 Haifa, Israel; 5 Department of Pathology, Kaplan Medical Center, Pasternak St. P.O.B. 1, 76100 Rehovot, Israel; 6 Sackler Faculty of Medicine, Tel-Aviv University, Ramat Aviv 69978, Tel-Aviv, Israel

**Keywords:** Hirschsprung's disease, Algorithm, Robustness, Machine learning, Digital pathology

## Abstract

**Background:**

Differences in the preparation, staining and scanning of digital pathology slides create significant pre-analytic variability. Algorithm-assisted tools must be able to contend with this variability in order to be applicable in clinical practice. In a previous study, a decision support algorithm was developed to assist in the diagnosis of Hirschsprung's disease. In the current study, we tested the robustness of this algorithm while assessing for pre-analytic factors which may affect its performance.

**Methods:**

The decision support algorithm was used on digital pathology slides obtained from four different medical centers (A-D) and scanned by three different scanner models (by Philips, Hamamatsu and 3DHISTECH). A total of 192 cases and 1782 slides were used in this study. RGB histograms were constructed to compare images from the various medical centers and scanner models and highlight the differences in color and contrast.

**Results:**

The algorithm was able to correctly identify ganglion cells in 99.2% of cases, from all medical centers (All scanned by the Philips slide scanner) as well as 95.5% and 100% of the slides scanned by the 3DHISTECH and Hamamatsu brand slide scanners, respectively. The total error rate for center D was lower than the other medical centers (3.9% vs 7.1%, 10.8% and 6% for centers A-C, respectively), the vast majority of errors being false positives (3.45% vs 0.45% false negatives). The other medical centers showed a higher rate of false negatives in relation to false positives (6.81% vs 0.29%, 9.8% vs 1.2% and 5.37% vs 0.63% for centers A-C, respectively). The total error rates for the Philips, Hamamatsu and 3DHISTECH brand scanners were 3.9%, 3.2% and 9.8%, respectively. RGB histograms demonstrated significant differences in pixel value distribution between the four medical centers, as well as between the 3DHISTECH brand scanner when compared to the Philips and Hamamatsu brand scanners.

**Conclusions:**

The results reported in this paper suggest that the algorithm-based decision support system has sufficient robustness to be applicable for clinical practice. In addition, the novel method used in its development – Hierarchial-Contexual Analysis (HCA) may be applicable to the development of algorithm-assisted tools in other diseases, for which available datasets are limited. Validation of any given algorithm-assisted support system should nonetheless include data from as many medical centers and scanner models as possible.

**Supplementary Information:**

The online version contains supplementary material available at 10.1186/s13000-024-01452-x.

## Background

Digital pathology is rapidly evolving as new technologies emerge, costs are reduced and availability increases. The practice of digital pathology most often involves obtaining a whole slide image (WSI) by digitally scanning a glass slide in one of many commercially available slide scanners. The scanned slides have been shown to be an adequate replacement to glass slides (in most instances) [1]. Consequently, many pathology departments have fully embraced the use of digital pathology for routine diagnosis [2–4]. Digital images have the added benefit of being readily available for use in computational pathology. These methods may include basic counting and measurements, as well as, more sophisticated tools based on artificial intelligence (AI) and deep learning (DL) [3]. DL tools have been successfully implemented in tasks such as tumor classification and grading, assessment of cellularity, mutation prediction and more [5–11]. More recently, similar tools have been used for algorithm-assisted [12, 13] or even fully-automated diagnosis [14, 15]. Different slide scanner models may have differences in both features and performance [16]. Different manufacturers use different file systems for WSI, which may not be easy to convert. This limitation may prove problematic, especially for large datasets obtained for either research or routine work at pathology departments who happen to make use of several different slide scanners [17]. Another issue is the possible loss of fidelity when compared to a glass slide due to a failure to detect and scan small tissue fragments or inconsistent image quality [18]. Furthermore, slides scanned by different scanners may appear different due to discrepancies in color. Standardization, validation and reproducibility of color for WSI is a well-known challenge, further complicated when using additional devices (scanner, display, etc.) with complex color transformations across devices and possible loss in color information [19]. Histological slides from different pathology departments differ not only in scanning but also in the preparation and staining of the original glass slides. As a result, their appearance may be highly heterogeneous (color, intensity, saturation, etc.). DL methods and algorithms may be highly sensitive to these differences as well as to artifacts, which an observant pathologist would not consider a problem. Relatively few studies attempted to evaluate the effects of histological artifacts on the performance of these algorithms [20–23]. As a result of these additional factors, it will often be wrong to simply assume that an algorithmic model or DL tool trained on slides from one department will be applicable to slides from another department, another scanner or even the same lab, without proper validation [24].

In a previous study a decision support algorithm (DSA) had been developed and used as part of a decision support system (DSS) meant to assist pathologists in the diagnosis of Hirschsprung's disease (HSCR) [12]. HSCR is a congenital disease characterized by an absence of ganglion cells in plexuses of the gastrointestinal tract. The histological diagnosis usually requires surveying dozens of slides (and possible use of immunostains) in search of ganglion cells, making the diagnosis of HSCR only in their absence. This process requires a significant investment of time and effort [25, 26].

Using an algorithm-assisted approach a pathologist with expertise in HSCR (7 years of experience) was able to achieve 100% accuracy with 95% time saved. A non-expert would have to send 20–58% of the cases to expert consultation to achieve similar performance [12]. Furthermore, in a follow-up study, a very short (10 min) training session with the same DSS was shown to greatly improve the pathologists performance in the algorithm-assisted diagnosis of HSCR while minimizing the need for expert consultation [13]. The DSS had been created based on a data set from a single hospital, processed at the same pathology laboratory and scanned by the same slide scanner model (Philips—IntelliSite Ultra-Fast Scanner).

In the current study, we aim to test and assess the robustness of the DSS by challenging the system to assist in the diagnosis of cases from other hospitals (slides from other pathology laboratories) scanned locally, as well as, on local cases, scanned at other facilities by different slide scanner models.

## Methods

All methods were performed in accordance with the relevant guidelines and regulations.

### Clinical samples

The cases used in the current study were obtained from four different medical centers as four distinct cohorts.

- Center A—Soroka university medical center in Be'er Sheva, Israel.
- Center B—Rambam medical center in Haifa, Israel.
- Center C—Emek Medical Center, Afula, Israel.
- Center D—Sourasky medical center, Tel-Aviv, Israel. The number of cases, slides and their usage are summarized in Table 1.

**Table 1:** Summary of the four cohorts of Medical centers A-D. Cases used for fine tuning of the DSA have been excluded from the final validation cohort

Cohort	Total number of cases	Total number of slides	Cases reserved for fine tuning of the DSA	Final validation cohort (cases)
Center A	51	396	2	49
Center B	31	143	2	29
Center C	51	322	2	49
Center D	59^a^	921	0	59
Total	133	1782	6	186

^a^Including the original validation cohort of 50 cases and 727 slides from a previous study

Pathological ground truth was determined in accordance with the medical records from each medical center. In cases where the available material was only partial (missing slides, infeasible recovery) the case was reviewed by an expert in HSCR (with 7 years of experience) and the pathological ground truth was based on the expert's opinion and applicable only to the sample in its current state. Therefore, if a sample was originally diagnosed as non-HSCR, based on the presence of ganglion cells within missing slides, it will be re-evaluated and treated as a HSCR case for the purpose of this study (As this classification has no direct bearing on the DSA's ability to identify ganglion cells).

### Algorithmic approach

The decision support system (DSS) used in this study had been developed as part of a previous study [12]. The DSS makes use of a decision support algorithm (DSA) based on a novel approach called "Hierarchical Contextual Analysis" (HCA). [12].

**Fig. 1:**
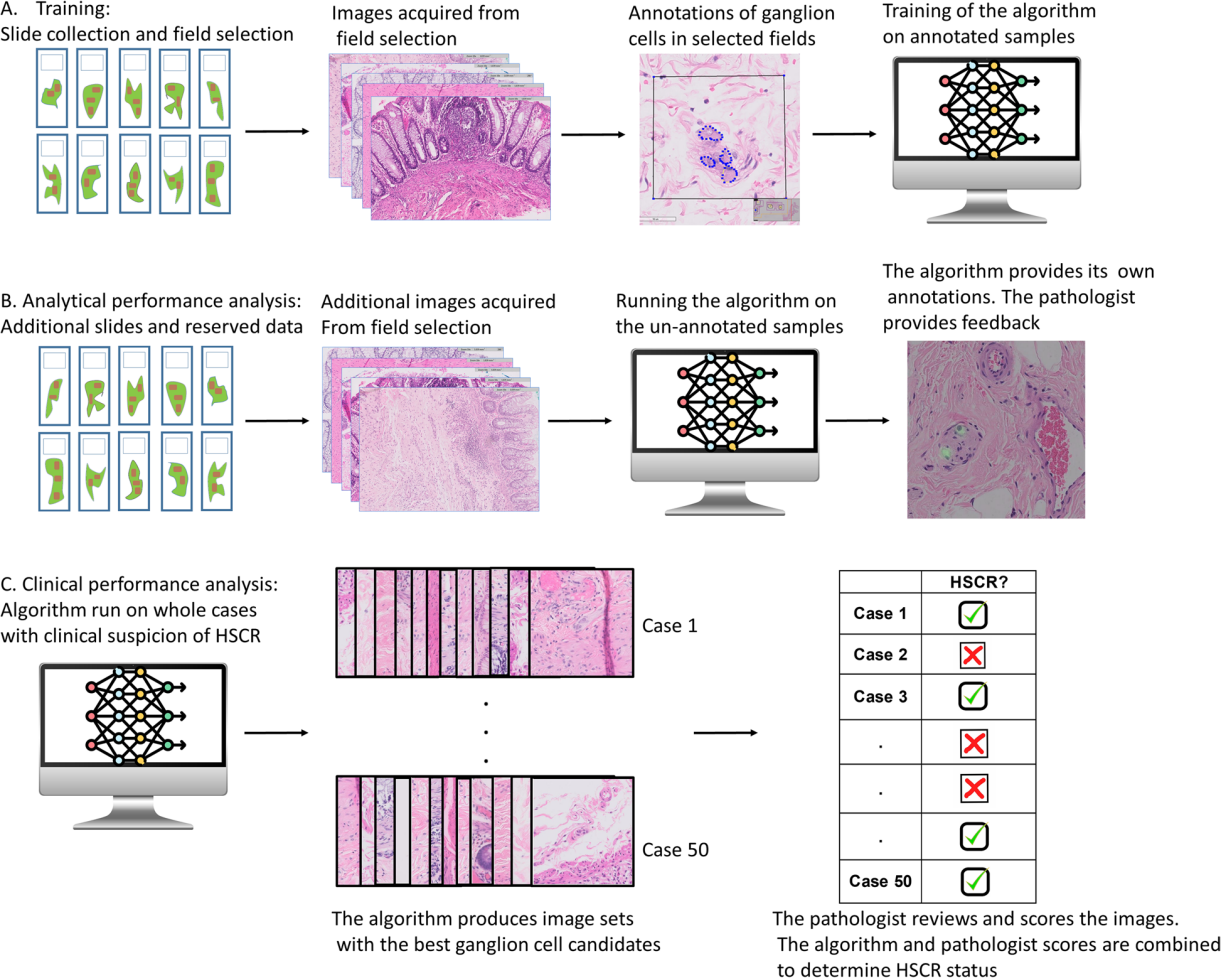
Algorithm construction and training schematic: **A** The algorithm training phase – Slides from normal colons were selected and ganglion cells were manually annotated The algorithm was then trained on these annotated fields. 10% of the data was reserved for further analysis. **B** The algorithm analytical performance phase—included reserved data, as well as additional slides from cases with clinical suspicion of HSCR. The algorithm was run on un-annotated data to produce annotations of its own, for which a pathologist has provided feedback. **C** The algorithm was then run on an additional 50 cases with clinical suspicion of HSCR and provided image sets of the best ganglion cell candidates it could find along with a their respective scores (0 to 1). The pathologist reviewed the image sets and provided his own score (1 to 5). The overall HSCR status of a given case was determined through a combination of the algorithm and pathologist scores (according to previously empirically determined decision criteria)

The model was developed in several stages. Initially raw un-annotated data was used along with insights derived from the pathologist's routine approach to HSCR in a phase of fully unsupervised learning aimed at creating the algorithm framework and degrees of freedom the algorithm possesses. The framework incorporated convolutional neural networks (CNN) along with decision processes inspired and based on expert knowledge. U-Net CNN structure was used as an approximation of the desired model. Subsequently, training of the algorithm was performed in several stages. Initially, WSIs of normal colonic tissue were used (86 in total). A single pathologist manually marked ganglion cells while unmarked fields were regarded as negative. A total of 3791 cells were marked. 10% of the data was reserved for performance measurement, while the reminder was used for the construction of the algorithm. We then tailored the system to provide its own annotations on 9 additional WSIs from cases with clinical suspicion of HSCR by training the deep neural networks. The pathologist provided feedback on each annotation, which was used to further fine tune the algorithm. Data augmentation was aimed at improving the robustness of the algorithm, in part via the use of generative adversarial networks (GANs). The algorithm was then run on data reserved for performance analysis and we determined the intersection over union (degree of overlap between the algorithm and the pathologist markings), the detection rate, and the rate of false alarms. Finally, the algorithm was run on a validation cohort of 50 cases with clinical suspicion of HSCR, containing 727 WSIs. The results have been published in a previous study [12]. A schematic description of this process is also depicted in Fig. 1.

### DSA basic functionality

The DSA segregates each digital slide into multiple images and surveys them for potential ganglion cell candidates. Each candidate is attributed a score between 0 and 1, indicating how closely it resembles a ganglion cell (With a "1" being a definitive ganglion cell). The images with the highest scores (closest to 1) are then presented to the pathologist (in sets of 3, up to 12 sets per case). The pathologist makes the diagnosis based on the presented image set, instead of having to manually survey dozens of whole slides, thus saving time and effort while maintaining diagnostic accuracy.

### Slide digitalization

The four cohorts of HSCR cases have been scanned at Center D, using the Philips—IntelliSite Ultra-Fast Scanner.

In addition, subsets of the Center D cohort have been scanned by two additional scanners: The Panoramic 250 flash III slide scanner, 3DHISTECH Ltd, Hungary and the Hamamatsu Nanozoomer S210, Hamamatsu Photonics, Japan.

**Fig. 2:**
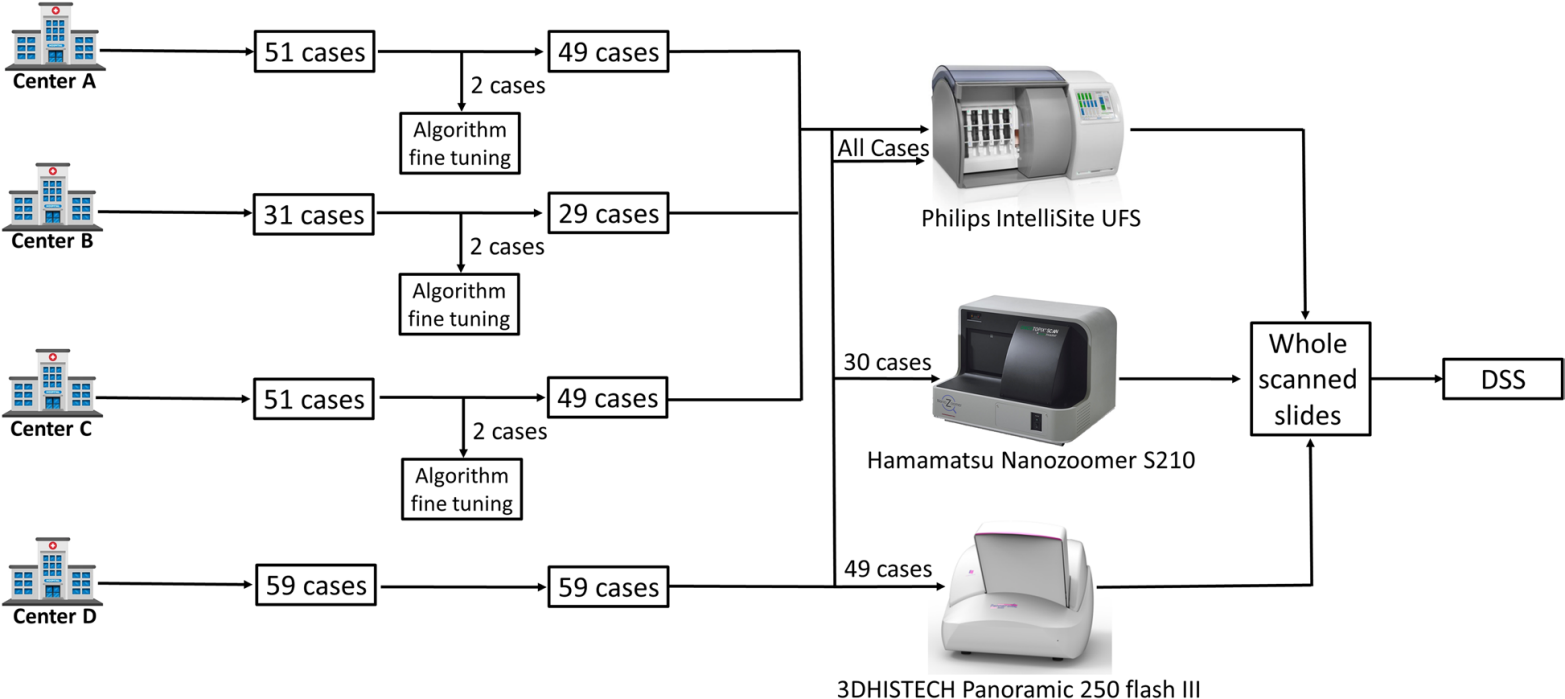
A schematic depicting the four cohorts, the number of cases used for fine-tuning of the algorithm and the distribution of cases between the three different scanner models. Two cases from medical centers **A-C** have been used for fine-tuning and therefore excluded from the validation cohort. All of the cases from medical centers **A-D** have been scanned by the Philips brand scanner. In addition, limited subsets (from the same cohort) of cases from medical center **D** have also been scanned by the Hamamatsu and 3DHISTECH brand scanners

Figure 2 summarizes the four cohorts and relevant subsets scanned by each scanner.

### Fine-tuning of the DSA

Two cases of each cohort from Centers A-C have been used for fine-tuning of the DSA (six cases in total). Center D was excluded from this phase, as the DSA had been originally trained on data from Center D. Manual segmentations of ganglion cells and background (negative samples) have been performed on each of the six cases used for fine-tuning and the data was integrated into the DSA in order to compensate for potential confounders such as inter-hospital differences in staining intensity, hue, sample processing etc. All of the segmentations were performed by a single pathologist (with 5 years of experience). A total of 337 definite ganglion cells have been marked (95, 184, 58 for Centers A, B and C, respectively). Additionally, 153 markings of findings suspicious for ganglion cells have been made (22, 103, 28 for Centers A, B and C, respectively)."Definite", "Suspicious" and "Negative" markings have all been used in the fine-tuning process. Cases used for fine-tuning of the algorithm have been excluded from the validation phase of the analysis. The final validation cohorts are therefore 49, 29 and 49 cases for Centers A, B and C, respectively. The fine-tuning process described here is merely the latest addition to the development and improvement of the DSA as described in previous works. The improvement in performance that could be attributed directly to this modification was modest, at less than 1%, and within the range of statistical error. For the purpose of this study, only the latest version of the DSA had been used, with previous versions considered part of the development process.

### Evaluation of the DSA

**Fig. 3:**
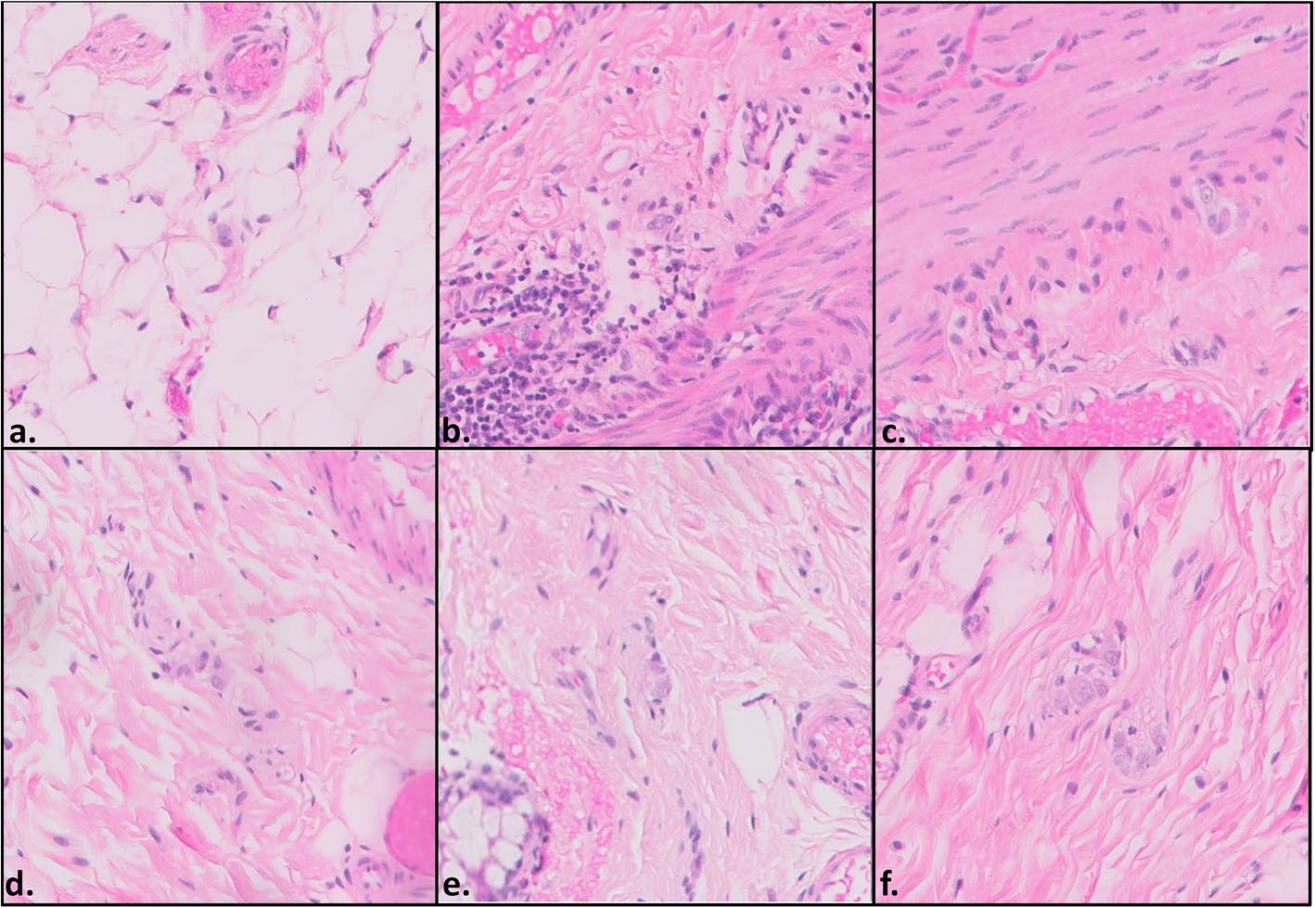
Microphotographs of ganglion cell candidates by their respective DSA scores in ascending order. The scores for each of the images a through f were 0.13, 0.32, 0.5, 0.62, 0.78 and 1, respectively. When examined by a pathologist, images **a** and **b** contained no ganglion cells (the low DSA score was appropriate), images **e** and **f** contained definitive ganglion cells (appropriate high DSA score), while images **c** and d were found to contain ganglion cells, yet their features were less pronounced (thus the intermediate DSA score)

The DSA reviewed all cases and extracted sets of images containing ganglion cell candidates, each is attributed a score between 0 and 1 (Fig. 3).

The total number of image sets was 675, 417, 465 and 667 for centers A through D, respectively. All of the extracted images have been reviewed by a single pathologist (with 5 years of experience) who was tasked with attributing a score between 1 and 5 to each image set.

The scores are as follows:

1. No ganglion cells seen (Certain),
2. No ganglion cells seen (Uncertain).
3. Uncertain/could not determine,
4. Ganglion cells seen (Uncertain).
5. Ganglion cells seen (Certain).

**Fig. 4:**
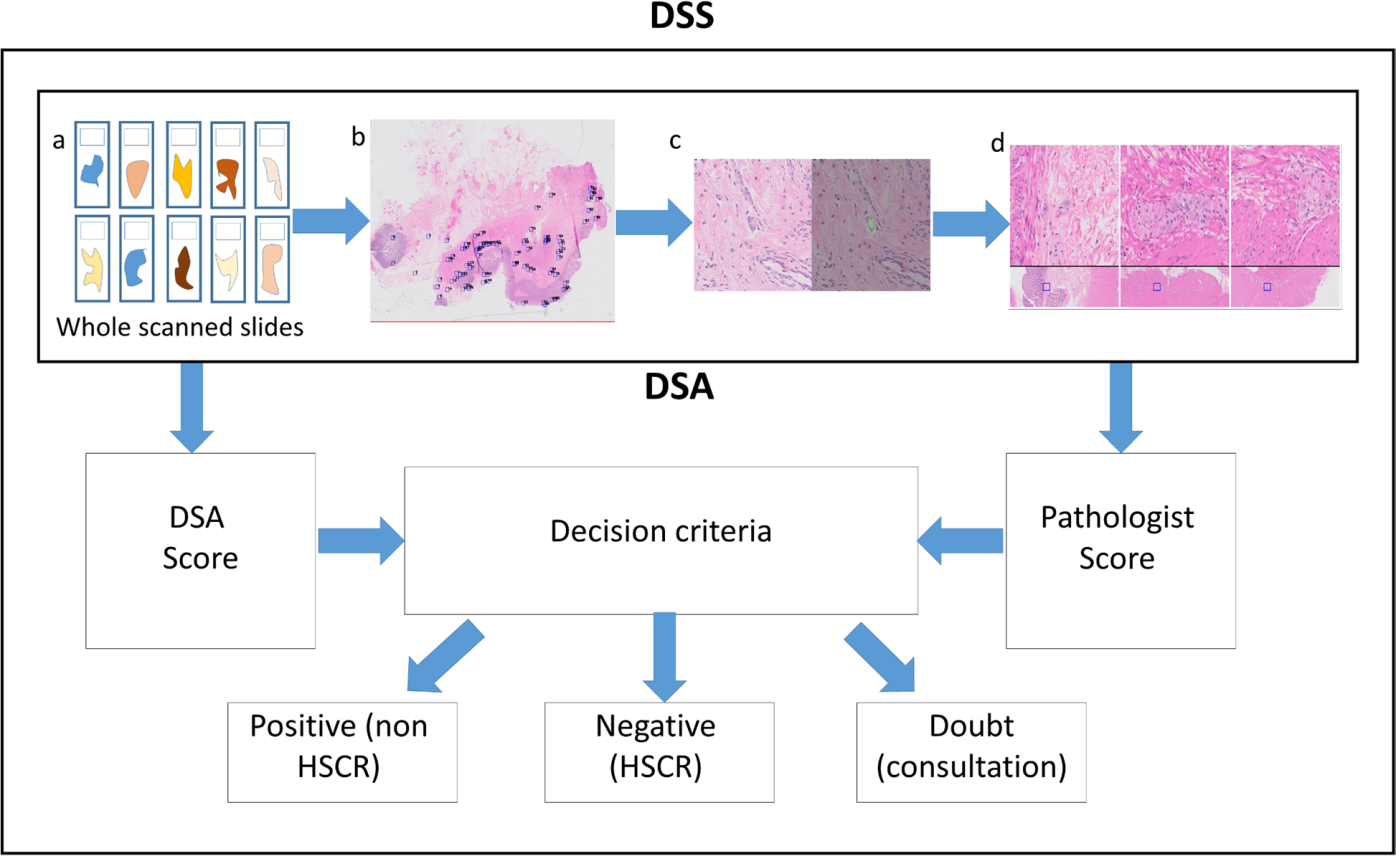
A diagram representing the structure of the DSS and the DSA, as well as the relationship between them. The process begins with digitally scanned whole tissue slides (**a**). The DSA searches through the scanned image and locates "areas of interest" which contain ganglion cell candidates (**b**). The DSA extracts images of each ganglion cell candidate and its immediate surroundings and also provides a score between 0 and 1 for each image, representing the level of "confidence" that the candidate is indeed a ganglion cell. The images containing the candidates with the highest scores are presented to the pathologist in up to 12 sets of 3 images each (**d**). The pathologist provides scores between 1 (no ganglion cells) and 5 (definite ganglion cells) to each image set. The function of the DSA is now complete. The DSS is in fact the combination of the pathologist score along with the DSA score. The DSS classifies each case according to a set of decision criteria as positive, negative or in doubt as follows: 1. Positive (non-HSCR)— the pathologist gave a score of 5 to any two (or more) image sets 2. Negative (HSCR)—The criterion for "Positive" is not met AND the average AI score is < 0.6. 3. In doubt (requires expert consultation)—The criterion for "Positive" is not met AND the average AI score is ≥ 0.6

The scores given by the pathologist are used along with the DSA scores (between 0 and 1) to determine the classification of each case according to criteria reported in a previous study [12]. Each case was classified as "positive" for ganglion cells, "negative" for ganglion cells or "in doubt", meaning a consultation from an expert HSCR pathologist is required (Fig. 4). Of note, "consultation" in this context means an "in house" revision of only the selected image sets (often requiring mere seconds and no more than a few minutes).

The classification made by the DSS (combining the DSA and pathologist) was then compared to the clinical and pathological diagnosis. For instance, a classification of "Positive" for ganglion cells, would only be considered as a "True positive" if it were to match with the pre-established diagnosis. Cases which were referred to expert consultation were reinterpreted as appropriate and their classification was assigned according to the diagnosis established by the expert.

### Further analysis

A more in-depth analysis followed to examine the algorithm's performance at the level of the image sets extracted from each case, rather than the case as a whole.

Special attention had been granted to image sets for which the pathologist and DSA scores showed complete discordance. The cut-offs set for this analysis were as follows:

A. Image sets to which the pathologist had attributed a score of "5" while the DSA score was below 0.3 ("False negative").
B. Image sets to which the pathologist had attributed a score of "1" while the DSA score was above 0.7 ("False positive").

The false negative and positive rates have been further analyzed for the probable cause for error. Several probable causes could apply to the same image set. The causative factors have been classified as:

1. Technical – errors related to slide preparation or digitization such as differences in staining quality, intensity, artifacts, low quality or out of focus scanning, etc.
2. Missing Specificity/Sensitivity – False negatives may be due to insufficient sensitivity, with the algorithm attributing a low score to a true ganglion cell. False positives may be due to insufficient specificity, with the algorithm attributing a high score to an item that resembles, yet is not a ganglion cell.
3. New/abnormal findings – errors related to the presence of tissues and entities the algorithm had not been exposed to during its creation and training. Examples may include normal tissue, such as squamous mucosa in the anal canal, or pathological findings such as heavy inflammation or adjacent tumoral tissue.
4. Unknown – errors for which no specific contributing factor could be defined.

### Color distribution analysis

To better understand the similarities and differences demonstrated in the performance of the DSA with different medical centers and scanner models, we explored basic differences in the behavior of the color distributions of the final images.

Differences in color and contrast were assessed separately for the various medical centers and different scanner models.

For the different medical centers, similar images were chosen from each. The images included similar features and structures. An RGB histogram was constructed for each image, depicting the percentage of pixels corresponding to each value in the R, G and B channels separately.

For the different scanner models, the assessment was conducted at the scale of both a single image and a complete case.

Single image level—images of the same area from the same slide (scanned by all three scanners) were compared. The images were chosen specifically due to having a disparity in color or contrast, apparent to a human observer. RGB histograms were constructed for each image.

Case level – two cases, which were scanned by all three scanners were chosen for the analysis. Each case included a total of 12 image sets with 3 images each. A RGB histogram was constructed based on the average distribution of each color channel pixel value of all 36 images from each case.

### Statistical analysis and metrics

A chi-square test of independence has been performed to compare rates and types of errors between the various medical centers and scanner models. Statistical significance was defined as *p* < *0.05*.

RGB histograms were compared using the L^2^ metric for Euclidean distance (squared) along with normalization of each histogram (to sum up to 1).

## Results

The DSS was able to correctly identify ganglion cells in nearly all of the cases, which were indeed positive for ganglion cells. In the cohort scanned at Center D, by the Philips IntelliSite Ultra-Fast Scanner, the DSS was able to correctly identify 39 out of 40 cases in which ganglion cells were indeed present (97.5%), with three cases requiring expert consultation. The DSS was able to correctly identify 100% of the cases positive for ganglion cells from Center A (28 cases, one referral), Center B (20 cases, no referrals) and Center C (37 cases, no referrals). When examining all cases, after referrals, only a single case out of 125 ganglion positive cases was misclassified, meaning 99.2% of all cases were correctly classified post referrals (compared to 96% pre-referrals). Of note, this is also the rate of correct identification for slides scanned by the Philips—IntelliSite Ultra-Fast Scanner. The total number of cases, presence or absence of disease and DSS performance for each cohort are summarized in Table 2.

**Table 2:** Summary of the number of cases, HSCR status and DSS performance metrics from each medical center

Cohort	Total number of cases	HSCR	non-HSCR	Correct classifications	Incorrect classifications	Referrals
Center A	49	21	28	49	0	1
Center B	29	9	20	29	0	0
Center C	49	12	37	49	0	0
Center D	59	20	39	58	1	3
Total (all centers)	186	62	124	185	1	4

When applied to slides scanned by different slide scanners, the DSS was able to correctly identify 100% of the ganglion cell positive cases which were scanned by the Hamamatsu Nanozoomer S210 slide scanner (30 cases, 2 referrals) and 95.5% of the ganglion cell positive cases which were scanned by the Panoramic 250 flash III slide scanner (21 out of 22 cases, 2 referrals).

Further analysis was conducted at the level of the image-sets instead of complete cases. False positives and false negatives (discordance between the pathologist and DSA scores) were analyzed with sub-classification of the probable causes for error.

The sub-classification for false positives and false negative is summarized in Table 3.

**Table 3:** Summary of total error, false negative and false positive rates for the four medical centers, and sub-classification of false positives and false negatives by possible causative factors (with % of the total errors of each type—false positives or false negatives)

Cohort	Image set total	Total errors (False positive and false negative)	Error type	Total (% of image set total)	Technical	Missing Specificity/Sensitivity	New/abnormal findings	Unknown
Center A	675	48 (7.1%)	False positive	2 (0.29%)	0 (0%)	0 (0%)	2 (100%)	0 (0%)
			False negative	46 (6.81%)	28 (60.9%)	17 (37%)	0 (0%)	1 (2.2%)
Center B	417	46 (11%)	False positive	5 (1.2%)	0 (0%)	5 (100%)	0 (0%)	0 (0%)
			False negative	41 (9.8%)	25 (61%)	16 (39%)	0 (0%)	0 (0%)
Center C	465	28 (6.0%)	False positive	3 (0.63%)	0 (0%)	3 (100%)	0 (0%)	0 (0%)
			False negative	25 (5.37%)	22 (88%)	2 (8%)	0 (0%)	1 (4%)
Center D	667	26 (3.9%)	False positive	23 (3.45%)	0 (0%)	20 (87%)	3 (13%)	0 (0%)
			False negative	3 (0.45%)	0 (0%)	3 (100%)	0 (0%)	0 (0%)

**Fig. 5:**
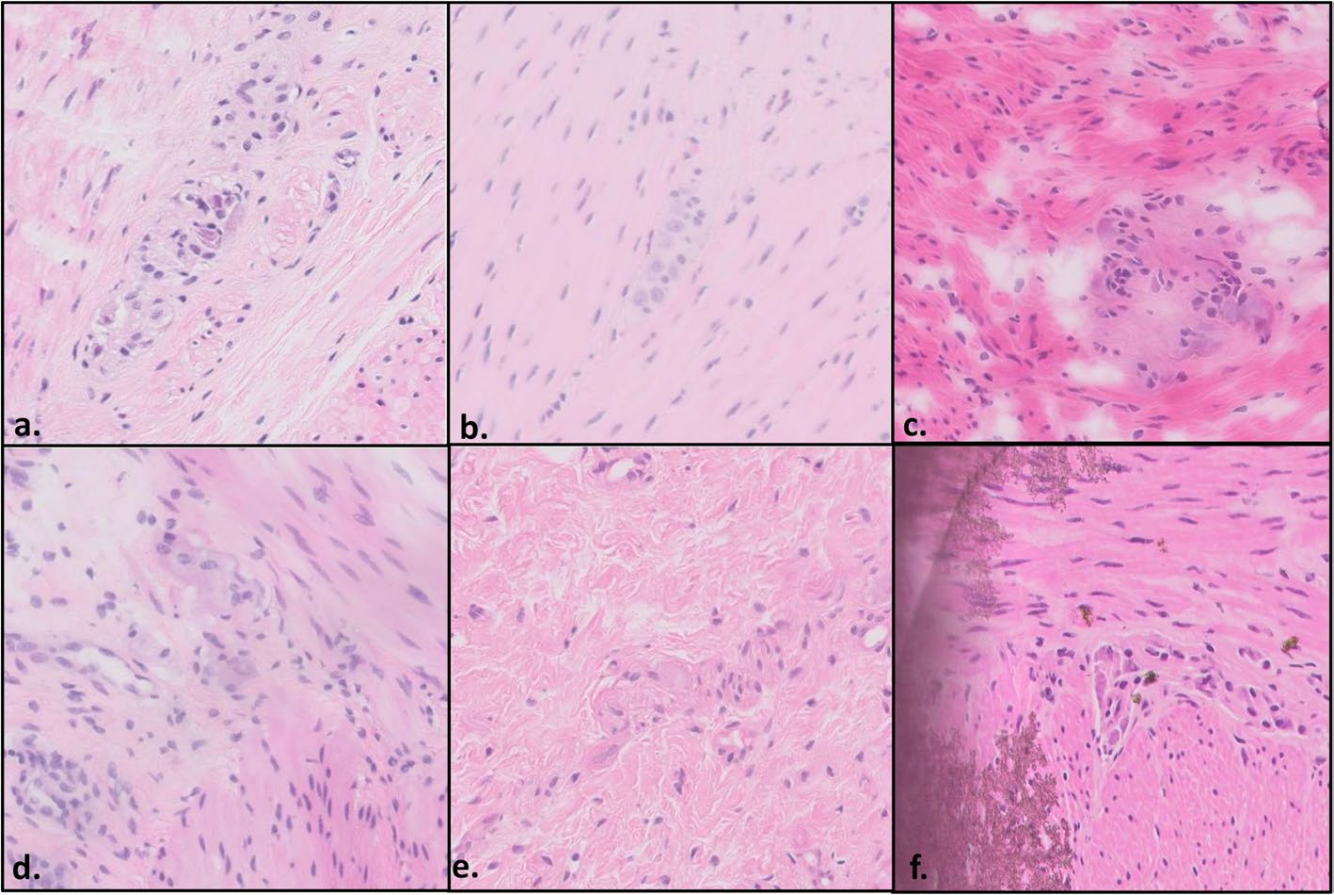
Several examples of images from Medical centers **A** (a,d), **B** (b,e) and **C** (c,f), which yielded a false negative result (DSA score ≤ 0.3, pathologist score = 5). The images include ganglion cells, however, confounding technical factors are present such as a faded image with low contrast (**a**, **b**, **e**), overstaining (**c**), poor resolution and focus (**d**) and artifactual changes (**f**)

The lowest total error rate was found in Center D (3.9%) compared to Centers A-C (7.1%, 10.8%, 6%, respectively,* p* = 0.00284). Of note, Center D provided the original data used for the construction and training of the algorithm. Interestingly, the vast majority of errors in Center D were false positives (3.45% vs 0.45% false negatives), while in the other centers the opposite was true with a higher rate of false negatives in relation to false positives (6.81% vs 0.29%, 9.8% vs 1.2% and 5.37% vs 0.63% for centers A-C, respectively,* p* < 0.0001). The relatively high number of false positives in Center D is attributed mainly to the cutoffs that were set for the DSA. The DSA is part of a decision support system and is designed to present the pathologist with the best ganglion cell candidates. Therefore, a false positive, which could often be easily dismissed by the pathologist, is not as concerning as the possibility of missing a true ganglion cell within the sample (a false negative). The cutoffs were adjusted appropriately, to favor sensitivity over specificity, and result in a greater proportion of false positives in relation to false negatives. However, the same cutoffs do not appear to be sufficient for the other medical centers, which differ in slide preparation and staining, resulting in lower sensitivity and a higher proportion of false negatives. Of note, most false negatives occurred on image sets with significant irregularities in staining intensity, contrast or focus, which were evident to the pathologist (Fig. 5). These changes did not prevent correct classification by the pathologist, yet appear to have a more pronounced effect on the DSA.

A similar analysis for false positives and false negatives has been performed for each slide scanner. The results are summarized in Table 4.

**Table 4:** Summary of total error, false negative and false positive rates for the three scanner brands, and sub-classification of false positives and false negatives by possible causative factors (with % of the total errors of each type—false positives or false negatives)

Scanner (by manufacturer)	Image set total	Total errors (False positive and false negative)	Error type	Total (% of image set total)	Technical	Missing Specificity/Sensitivity	New/abnormal findings	Unknown
Hamamatsu Photonics	348	11 (3.2%)	False positive	3 (0.9%)	2 (66.7%)	1 (33.3%)	0 (0%)	0 (0%)
			False negative	8 (2.3%)	0 (0%)	7 (87.5%)	1 (12.5%)	0 (0%)
3D Histech	447	44 (9.8%)	False positive	40 (8.9%)	7 (17.5%)	10 (25%)	0 (0%)	23 (57.5%)
			False negative	4 (0.9%)	0 (0%)	4 (100%)	0 (0%)	0 (0%)
Philips	667	26 (3.9%)	False positive	23 (3.45%)	0 (0%)	20 (87%)	3 (13%)	0 (0%)
			False negative	3 (0.45%)	0 (0%)	3 (100%)	0 (0%)	0 (0%)

The total error rate was similar between the Philips and Hamamatsu brand slide scanners. The 3DHISTECH brand scanner showed a higher total error rate (9.8%) compared to the Hamamatsu (3.2%,* p* = 0.00023) and Philips (3.9%, *p* = 0.000061) brand scanners. However, the rate of false positives and false negatives for the 3DHISTECH and Philips brand slide scanners were similar and not statistically significant (*p* = 0.074). The Hamamatsu brand scanner had a higher rate of false negatives but the total number of errors was low and the difference in absolute numbers is small.

When comparing the effects of the different medical centers and scanners on the error rates of the DSA, the difference in total error rates was not statistically significant. However, errors associated with different medical centers were mainly false negatives, whereas errors associated with different scanners were mainly false positives (*p* < 0.00001). This suggests that training of the DSS with data from additional medical centers may contribute to its sensitivity and aid in reducing false negatives. On the other hand, data from different scanners may help improve the specificity of the DSA and reducing false positives.

**Fig. 6:**
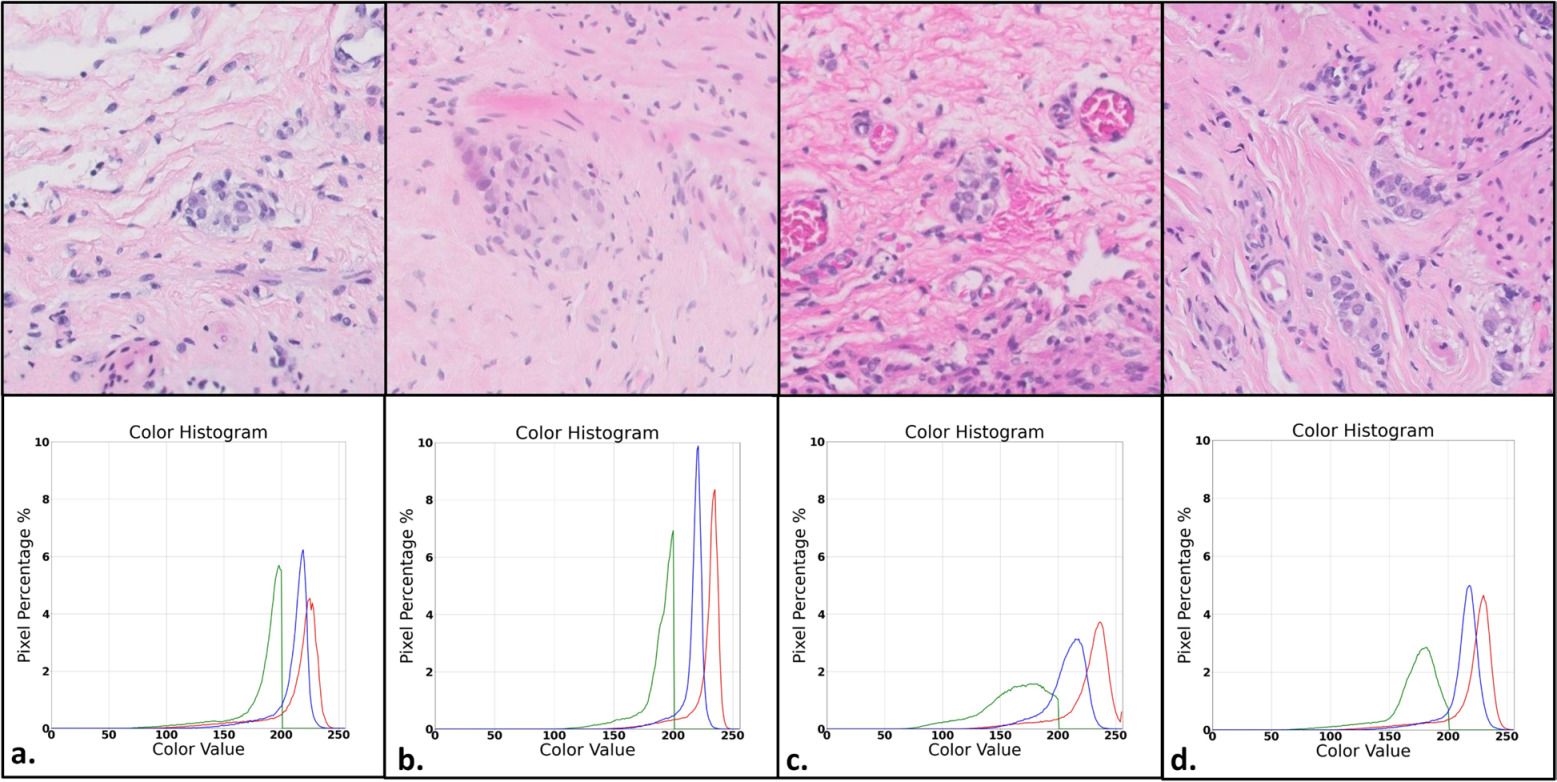
Microphotographs of ganglion cells and their immediate surroundings from each medical center along with RGB histograms. The images demonstrate differences in color, resolution and texture with corresponding differences in each color channel pixel value distribution as demonstrated in the histograms (expressed as the percentage of pixels corresponding to each pixel value). Each set of image and histogram "a" through "d" corresponds to the different medical centers A through D, respectively

**Fig. 7:**
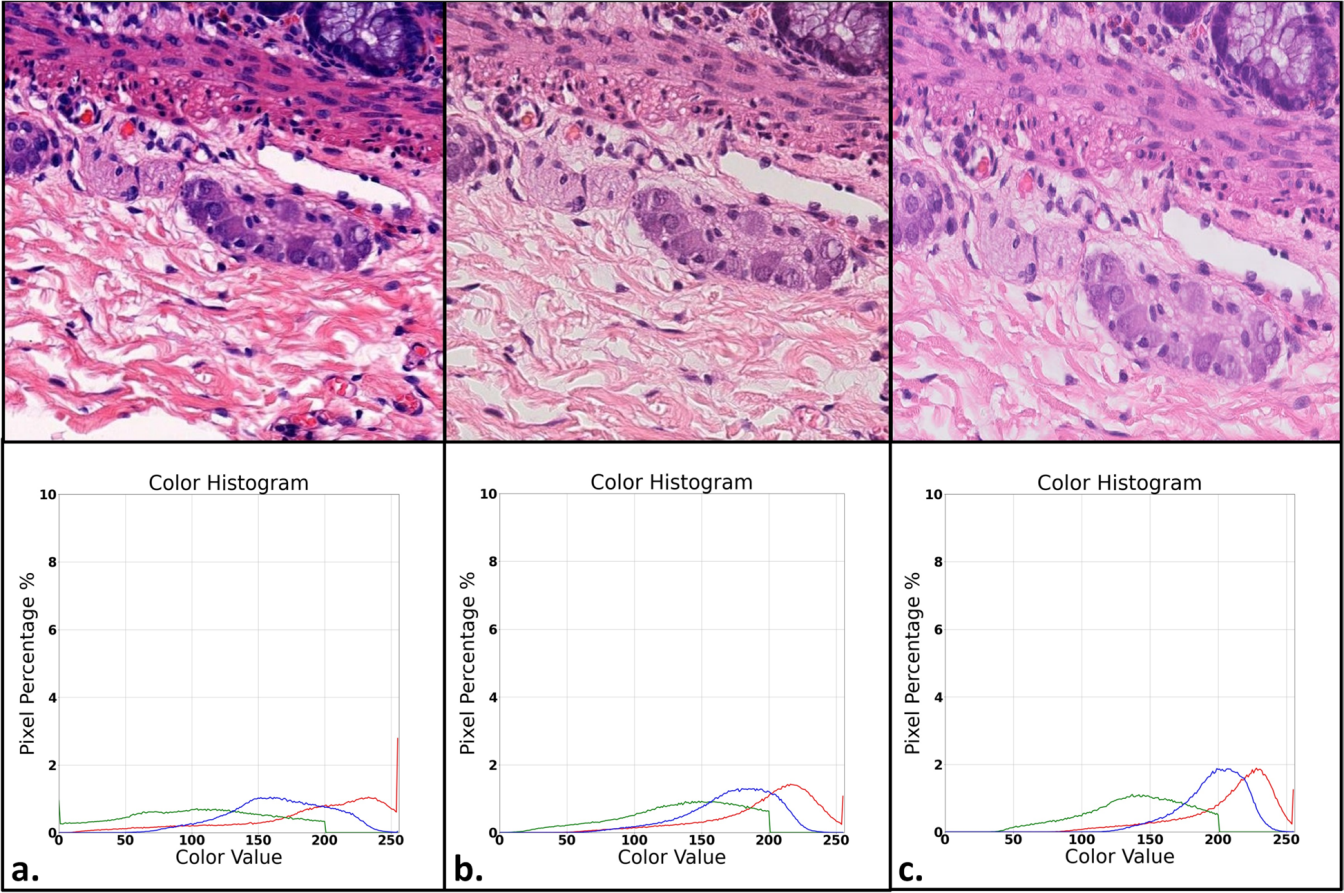
Microphotographs of the same ganglion cell containing area, scanned at each of the three different scanners, along with RGB histograms. The images demonstrate differences in contrast, color resolution and texture with corresponding differences in each color channel pixel value as demonstrated in the histograms (expressed as the percentage of pixels corresponding to each pixel value). Image and histogram set "a" corresponds to the Panoramic 250 flash III slide scanner (3DHISTECH), set "b" corresponds to the Nanozoomer S210 (Hamamatsu), set "c" corresponds to the IntelliSite Ultra-Fast Scanner (Philips)

**Fig. 8:**
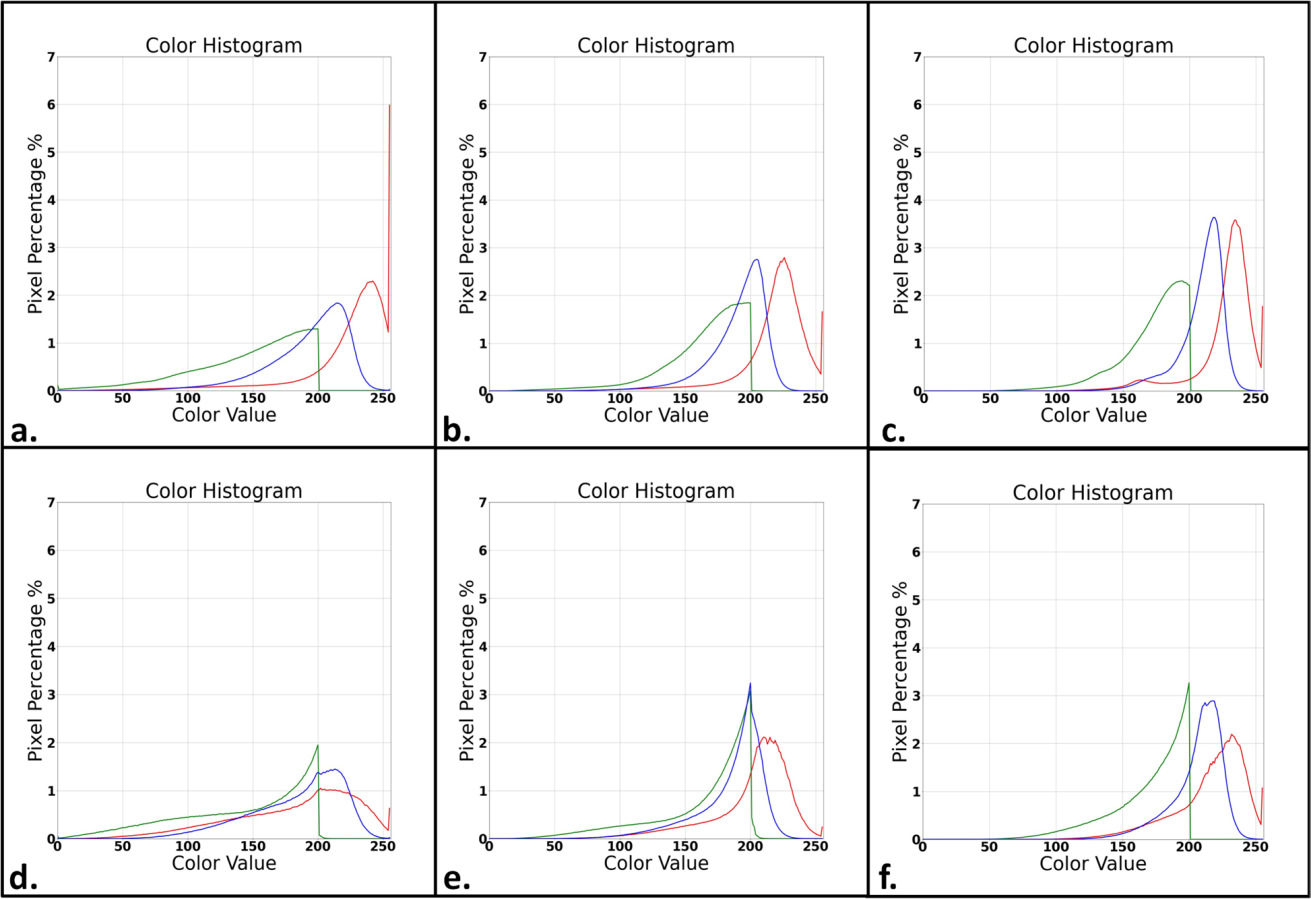
RGB histograms based on the average color pixel value distribution of 36 images from two cases, each scanned by three different slide scanners: the 3DHISTECH Panoramic 250 flash III slide scanner (histograms a and d), the Hammatsu Nanozoomer S210 (histograms b and e) and the Philips IntelliSite Ultra-Fast Scanner (histograms c and f). Greater similarities are noted between the Hamamatsu and Philips slide scanners, with peaks at similar color pixel values and relatively small variance in amplitudes. The 3DHISTECH slide scanner shows a greater distribution of color pixel values with lower peaks, accordingly

Inspection of images of similar elements (ganglion cells, nerves, muscles, etc.) from each medical center, revealed significant differences in color, resolution and texture, apparent even to the naked eye. An RGB distribution histogram further demonstrated the differences in color distribution, possibly due to differences in processing and staining between the four medical centers (Fig. 6). A similar analysis has also been performed for the different scanners on a single slide (Fig. 7) as well as on two whole cases (Fig. 8) with differences likely attributed to differences in resolution, contrast and scanning protocols.

Among the different medical centers, the image from Center B showed the narrowest distribution with high and narrow peaks towards the higher end of each color channel pixel value. The image from Center C showed the greatest distribution with lower peaks and a greater pixel value range. Note, however, that the images were chosen to include similar elements but are not from the same slides. Therefore, the specific elements present and their relative quantity in each image may have a significant effect on RGB distribution, in addition to any effects attributed to differences in staining and slide preparation.

When examining the RGB histograms for the different scanners, generally, images scanned by the Hamamatsu and Philips brand scanners showed relatively similar distributions of each color channel pixel value, while the 3DHISTECH slide scanner trended towards a greater distribution of color values than the other scanners, with relatively reduced "peaks", meaning there was a greater variance in color pixel values with less pixels at each specific value. This pattern was evident when comparing a single image of the same area from a single slide (scanned on each of the scanners) and was even more pronounced when examining the average distribution of all 36 images for an entire case. Calculation of the Euclidean distance between the histograms revealed a Euclidean distance of 0.0040 between the Philips and Hamamatsu brand scanners, versus a Euclidean distance of 0.0074 (85% greater) between the Philips and 3DHISTECH brand scanners. These metrics further demonstrate the observable differences between the RGB histograms of the different scanner brands, as described.

## Discussion

Decision support systems and algorithms used in digital pathology require a certain degree of robustness and generalizability in order to be appropriate for clinical use. Demonstration of this robustness usually requires validation on datasets obtained from multiple centers. Validation is necessary in order to account for pre-analytical sources of variation, such as tissue handling, slide preparation, staining, scanner model and scanning protocol [27–31]. The variation in staining and fixation between different laboratories may be significant even if the internal quality control of each center is maintained. These differences must be accounted for during the development and validation of the algorithm [32]. The digitization process is also subject to pre-analytic sources of variation such as scanner-to-scanner differences in color calibration, image resolution, focus and magnification. These differences have been shown to have an observable impact on algorithms applied to digital slides [33]. The amount and variability of the data provided during the development of the algorithm should be representative of the data encountered in clinical practice. The number of medical centers or different scanners required to achieve this representability depends on the diagnostic question and has been poorly explored in the literature [34, 35]. Limited generalizability remains one of, if not the most important hurdle limiting the implementation of AI-based support systems in clinical practice [36]. Several methods have been employed to account and correct for these variations. One approach is to introduce as much variability as possible in the provided data, but availability may be limited. It is also possible to introduce artificial variability through color augmentation meant to mimic staining differences between different laboratories [20, 36]. A different approach is normalization of the images to a common standard, an approach that was shown to improve performance even for algorithms developed on a limited dataset. Normalization may be conducted by several different methods, including histogram-based color matching, normalization after stain separation and style transfer via neural network [37]. However, data derived from stains is stored in the combination of the three RGB channels. Therefore, normalization may cause distortions in the signals [38]. Furthermore, this approach generally requires applying the normalization process to each target image and may be costly in time and computational resources [39, 40]. AI methods may also assist in this process with tools designed to normalize stain and color [40–42] or provide more comprehensive quality control and standardization [43, 44].

The current study aimed to assess the robustness of a previously developed DSS designed to assist a pathologist in the diagnosis of HSCR. However, HSCR is a rare disease with a worldwide incidence ranging from 1:5000 and 1:10,000 live births [45]. Normally, the development of a deep learning algorithm with clinically useful performance would require a large dataset, especially considering the need to account for the many sources of pre-analytic variation. Some researchers have even opted for especially large cohorts comprised of tens of thousands of slides [7]. When dealing with rare diseases, this approach is far less feasible. Augmentation of the images or normalization can reduce the requirement, yet are insufficient on their own. For this reason, the algorithm (DSA) used in the DSS which was employed in the current study, had been developed using a novel approach: "Hierarchical Contextual Analysis" (HCA) [12]. HCA attempts to mimic the way in which a pathologist examines a given tissue (in this instance, colon) and makes the diagnosis (in this instance, HSCR). The "Hierarchical" component relates to the relative location and orientation of a given finding and their meaning. For example, a ganglion cell candidate detected in the epithelial layer would be excluded, as the algorithm determines its location and crosses this information with the previously "learned" fact that the epithelial layer does not contain ganglion cells. The "Contextual" component relates to the immediate surroundings of a given finding. For instance, the fact that ganglion cells tend to appear in clusters and along with nerve fibers is considered when assessing any given ganglion cell candidate [12]. Using HCA allowed for the construction of a potent algorithm-assisted tool from a limited dataset. It is imperative to note, that the DSS is meant to assist and not replace the pathologist. The use of algorithm-assisted tools has been shown to decrease human error and provide better overall performance than either the pathologist or the algorithm alone [46, 47].

In the current study, we aimed to assess the robustness of the same algorithm by applying it to datasets from different medical centers and scanned with different slide scanners. All of the data used to construct the original algorithm had been from a single medical center with only a case-by-case variation to contend with. No AI or other computational tools were used to introduce artificial variance or perform any significant normalization of the dataset. Before attempting to assess the algorithm, additional data, from the other medical centers was used. However, only two cases from each additional medical center had been reviewed and integrated into the algorithm to account for the variability in staining, fixation or any other factors introduced. All slides, from all medical centers were scanned on a single scanner model (Philips—IntelliSite Ultra-Fast Scanner). Despite this extremely limited addition of data, the algorithm and by extension the DSS was able to correctly identify all of the cases, from medical centers A-C, which were positive for ganglion cells with 100% sensitivity (For center D the sensitivity was 97.5%). Moreover, no additional data and no form of normalization or correction have been applied (externally) to the slides scanned by the Panoramic 250 flash III and Hamamatsu Nanozoomer slide scanner, and yet, the algorithm was able to identify all of the cases which were positive for ganglion cells with 100% sensitivity. Of note, the current study only examined slides which were either created in a different laboratory or scanned with a different scanner model (using locally produced slides), but none with both parameters. Future works should include cases from different centers, which were also scanned by a different scanner model.

An additional, more in-depth analysis has been performed at the level of the image sets rather than complete cases. The analysis included the rate and possible causes of false positive and false negative results for each medical center and scanner included in this study. While the current study was not designed for a thorough analysis on an image-to-image basis, the results highlight several trends.

False negatives results were most commonly attributed to "technical" factors, across all four medical centers and regardless of the scanner model used. Factors such as differences in staining intensity, color, artifacts and scanning resolution and focus, appear to affect the DSA's ability to correctly identify a ganglion cell, to a greater degree than these same factors might affect a trained human observer [48]. Additional samples for training and validation of the DSA may further improve its performance and minimize the effects of technical factors.

Center D showed the lowest rate of false negatives among all medical centers. This trend was expected, as the DSS was created and trained on data from Center D, and therefore would be expected to perform better under the same conditions. Artifactual changes common to Center D will have been presented to the DSS during its past training and will be more easily ignored, when compared to similar changes from other medical centers, which may be accompanied by additional differences in processing, staining and slide preparation.

Additionally, the DSS appears to display "overconfidence" when attributing scores to image sets from Center D, with higher overall scores attributed to the images (compared to the other centers) including images which were negative for ganglion cells, resulting in a relatively higher rate of false positives. It should be noted however, that such false positives are easily dismissed by a trained pathologist. The purpose of the DSS is to find and present the best ganglion cell candidates to the pathologist, it is therefore under-representation and failure to present a false negative which may result in failure to identify a ganglion cell present in the sample, while over-representation and the inclusion of false positives should have no bearing on the final diagnosis made by the pathologist.

Images obtained from the different medical centers demonstrated differences in staining intensity and contrast, often at a degree noticeable by a human observer (pathologist). RGB histograms comparing similar images between the four medical centers demonstrated significant variability in color pixel values for all three RGB channels.

The analysis of image sets obtained from the three different scanner brands revealed a higher total error rate for the 3DHISTECH brand scanner when compared to the Hamamatsu and Philips brand scanners. However, the rates of false positives and false negatives was similar for both the Philips and 3DHISTECH brand scanners, as opposed to the great disparity in the relative rates of false positives and false negatives seen with the different medical centers. The results suggest that the DSA is more sensitive to differences in slide preparation and staining than to differences resulting from the scanning and digitization process employed by each scanner model.

RGB histograms comparing a specific area from a single slide among all three scanners were constructed and demonstrate a similar distribution of each color channel pixel value for the Philips and Hamamatsu brand slide scanners, while the 3DHISTECH brand slide scanner showed a greater distribution of color pixel values (less pixels at each pixel value, with a wider range of pixel values) for all three RGB channels. The exact cause of these differences between images produced by the different scanner models is likely technical, yet beyond the scope and aim of the current study. We are, however, able to confirm that such differences are present and are significant enough to be reflected in the final images as well as within the RGB histograms, including significant differences in the Euclidean distance when comparing the histograms for the Philips brand scanner to those of the Hamamatsu and 3DHISTECH brand scanners.

## Conclusions

The results of this current study are suggestive of the robustness of the algorithm and demonstrate the strength of HCA as a method to create powerful, effective and robust algorithm-assisted decision support systems even with a limited data set. HCA and similar techniques may prove invaluable for the development of algorithms involving rare diseases, for which quality data is inherently limited. Nonetheless, we recommend including data from as many different laboratories and scanner models as possible as part of the validation process of any given algorithm-based decision support system. Further research would be required to establish the applicability of HCA with shifting domains and classifiers.

Additionally, our results suggest that data from various medical centers would likely provide a great contribution towards reducing the rate of false negatives whereas data from different scanners may assist in slightly reducing false positives. Further studies should evaluate these observations in larger cohorts and different use cases.

### Supplementary Information

**Additional file 1: Supplementary table 1.** Summary of the total error rates, false positives and false negatives between Center D using the Philips brand scanner and the other medical centers (slides scanned by the Philips brand scanner) and the other scanners (withslides from Center D).

## Data Availability

The datasets used and/or analyzed during the current study are available from the corresponding author on reasonable request, pending institutional review board approval.

## Abbreviations

AI: Artificial Intelligence
DL: Deep Learning
DSS: Decision Support System
DSA: Decision support Algorithm
HCA: Hierarchial-Contexual Analysis
HSCR: Hirschsprung (disease)
WSI: Whole Slide Image
